# Sex differences in outcomes and risk factors among elderly patients with ischemic stroke

**DOI:** 10.18632/oncotarget.21967

**Published:** 2017-10-19

**Authors:** Chunying Zou, Chunjie Wei, Zengmian Wang, Yuling Jin

**Affiliations:** ^1^ Department of Neurology, The First Affiliated Hospital of Jiamusi University, Heilongjiang, 154002, China

**Keywords:** atherosclerotic stroke, sex differences, outcomes, elderly

## Abstract

We aimed to investigate the sex differences in the clinical characteristics and risk factors for adverse outcomes among elderly patients with atherosclerotic stroke. We recruited 942 consecutive patients with atherosclerotic stroke aged 75 years and older between January 2008 and December 2013 from Jiamusi University First Hospital, China. Stroke subtype, severity, risk factors, and outcomes (mortality, dependency, and recurrence) at 3 and 12 months after stroke were recorded and assessed. Mortality at 3 months after stroke was higher in men than in women. Stroke severity was an independent risk factor for mortality, dependency, and recurrence at 3 and 12 months after stroke in both men and women. However, the presence of total anterior circulation infarct and obesity protected against mortality at 3 months after stroke in men, while total anterior circulation infarct was a risk factor for dependency at 3 months after stroke in women. In women, positive associations were found between fasting plasma glucose level and mortality at 3 months after stroke and between hypertension, atrial fibrillation, and recurrence at 12 months after stroke. These findings suggest that it is crucial to control the primary risk factors individually by sex, especially regarding hypertension and atrial fibrillation management, to improve secondary prevention of stroke among the elderly and reduce the burden of stroke in China.

## INTRODUCTION

Stroke was the second most common cause of death and the third most common cause of reduced disability-adjusted life-years worldwide in 2010 [1]. Stroke incidence has declined in industrialized countries over the last 30 years, but it has recently become the leading cause of death in rural areas and the third cause of death in urban areas in China [2–4]. Aging is the most important non-modifiable risk factor for stroke. At present, more than half of all strokes occur in people over 75 years of age [5]. The case-fatality rates are higher in the very old; among sur*vivo*rs, post-stroke functional status is worse, in both the short-term and the long-term [6–11].

Studies have shown that the prevalence of stroke in women is lower than that in men [12–15], but several other studies have shown that stroke rates and mortality are higher among older women than among older men [16–18]. However, few studies have examined sex differences in the clinical characteristics and risk factors for adverse outcomes in patients with atherosclerotic stroke.

Thus, we aimed to investigate the sex differences in the clinical characteristics and risk factors for adverse outcomes in patients aged 75 years and older with atherosclerotic stroke.

## RESULTS

### Sex differences in clinical characteristics and risk factors among patients with atherosclerotic stroke

Total of 1070 patients aged 75 years and older with AIS during the study periods, patients accounted for 67.6% for LAA (68.3% in men and 66.7% in women, *P* = 0.578) , 20.5% for SAO (20.8% in men and 20.1% in women, *P* = 0.065) , 10.5% for CE (9.3% in men and 12.0% in women, *P* = 0.158), 0.7% for unknown causes (0.7% in men and 0.6% in women, *P* = 1.000), and 0.8% for others (1.0% in men and 0.6% in women, *P* = 1.000).

This study included 942 stroke patients aged 75 years and older. Of these patients, there were 536 men (56.9%) and 406 women (43.1%). The mean age was 79.59 years overall, and 79.72 years for men and 79.42 years for women (*P* > 0.05). There were no significant sex differences in the Oxfordshire Community Stroke Program (OCSP) classification and stroke severity. The prevalences of hypertension and obesity were higher in women than in men, but the opposite pattern was observed in the prevalences of current smoking and alcohol drinking. The total cholesterol (TC), triglycerides (TG), and low-density lipoprotein cholesterol (LDL-C) levels were higher in women than in men (Table 1).

**Table 1:** Sex differences in clinical characteristics and risk factors among patients with atherosclerotic stroke

Characteristics	Total	Men	Women	*P*
Case, *n* (%)	942	536 (56.9)	406 (43.1)	—
Age, years, means (SD)	79.59 (3.97)	79.72 (3.95)	79.42 (4.00)	0.254
OCSP Classification, *n* (%):				0.678
TAPI	60 (6.4)	35 (6.5)	25 (6.2)	
PACI	534 (56.8)	295 (55.1)	239 (59.0)	
LACI	66 (7.0)	38 (7.1)	28 (6.9)	
POCI	280 (29.8)	167 (31.2)	113 (27.9)	
Stroke severity:				0.251
Mild	555 (58.9)	328 (61.2)	227 (55.9)	
Moderate	278 (29.5)	148 (27.6)	130 (32.0)	
Severe	109 (11.6)	60 (11.2)	49 (12.1)	
Neurological function^*^:				
NIHSS	8.0 (9)	7.5 (9)	8.0 (9)	0.065
BI	35.0 (45)	45.0 (45)	27.5 (40)	0.005
mRS	4.0 (1)	4.0 (1)	4.0 (1)	0.125
Risk factors, *n* (%):				
Hypertension	698 (74.1)	376 (70.1)	322 (79.3)	0.001
Diabetes	280 (29.7)	148 (27.6)	132 (32.5)	0.103
Atrial fibrillation	80 (8.5)	44 (8.2)	36 (8.9)	0.720
Obesity	132 (14.0)	58 (10.8)	74 (18.2)	0.001
Current smoking	204 (21.7)	152 (28.4)	52 (12.8)	< 0.001
Alcohol drinking	71 (7.5)	69 (12.9)	2 (0.5)	< 0.001
Fasting measurements, means (SD), mmol/L:				
FPG	6.49 (2.51)	6.41 (2.39)	6.59 (2.68)	0.363
TC	4.83 (1.08)	4.57 (0.97)	5.19 (1.11)	< 0.001
TG	1.31 (0.88)	1.22 (0.63)	1.43 (1.12)	< 0.001
HDL-C	1.13 (0.60)	1.10 (0.75)	1.16 (0.30)	0.153
LDL-C	3.00 (0.87)	2.83 (0.77)	3.22 (0.96)	< 0.001

^*^ indicated that data were presented as median with interquartile range.

### Sex differences in outcomes at 3 and 12 months after stroke

The mortality, dependency, and recurrence rates at 3 months after stroke were 12.3%, 38.2%, and 8.6% in men; the corresponding rates were 7.8%, 44.8%, and 7.7% in women, respectively. Mortality at 3 months after stroke was significantly higher in men than in women (12.3% vs 7.8%, *P* = 0.030). There were no significant sex differences in mortality at 12 months after stroke or in dependency and recurrence at 3 and 12 months after stroke (Table 2).

**Table 2:** Sex differences in clinical characteristics and risk factors among patients with atherosclerotic stroke in the univariate analysis

Characteristics	Men	Women	*P*
3 Months after stroke:			
Mortality	64 (12.3)	30 (7.8)	0.030
Dependency	174 (38.2)	158 (44.8)	0.058
Recurrence	41 (8.6)	28 (7.7)	0.666
12 Months after stroke:			
Mortality	85 (19.7)	47 (14.6)	0.067
Dependency	123 (35.3)	102 (36.8)	0.702
Recurrence	99 (25.1)	69 (22.7)	0.468

**Figure 1:**
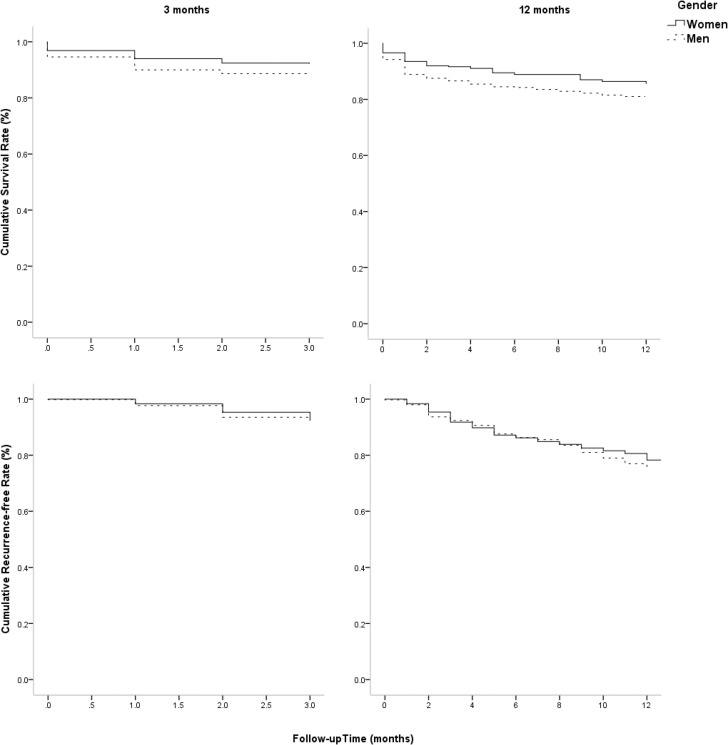
Sex differences in survival rate and recurrence-free rate at 3 and 12 months after stroke Figure 1 showed that there was a higher risk of mortality in male patients than in female patients at 3 months after stroke, with log-rank of 4.751 (*P* = 0.029) using Kaplan-Meier. However, there were not sex differences in mortality at 12 months and recurrence at 3, 12 months after stroke among elderly patients with atherosclerotic infarction (all *P* > 0.05).

Figure 1 showed that there was a higher risk of mortality in male patients than in female patients at 3 months after stroke, with log-rank of 4.751 (*P* = 0.029) using Kaplan-Meier. However, there were not sex differences in mortality at 12 months and recurrence at 3, 12 months after stroke among elderly patients with atherosclerotic infarction (all *P* > 0.05).

### Risk factors for adverse outcomes at 3 and 12 months after stroke by sex in the univariate analysis

Overall, sex, age, stroke severity, obesity, and fasting plasma glucose (FPG) level were associated with mortality at 3 months after stroke; age, stroke severity, and FPG level were also associated with mortality at 12 months after stroke. Stroke subtype, stroke severity, obesity, and LDL-C and FPG levels were associated with dependency at 3 months after stroke; stroke subtype, stroke severity, and alcohol consumption were strongly associated with dependency at 12 months after stroke. However, recurrence was only associated with stroke severity (Table 3, Table 4).

**Table 3:** Univariate analysis for risk factors of outcomes at 3 months after stroke

Factors	Mortality			Dependency			Recurrence		
	Men	Women	Total	Men	Women	Total	Men	Women	Total
Age:									
Yes	80.52 (4.17)	80.60 (5.27)	80.54(4.52)^*^	79.59 (3.70)	80.17 (4.46)^*^	79.52 (3.78)	79.90 (4.12)	79.44 (3.87)	79.54 (3.72)
No	79.58 (3.89)	79.09 (3.70)	79.37 (3.81)	79.58 (4.01)	78.92 (3.66)	79.26 (3.84)	79.62 (3.90)	78.81 (3.55)	79.40 (3.87)
OCSP:									
TACI	13 (37.1)^*^	6 (30.0)^*^	19 (34.5)^*^	14 (63.6)^*^	9 (64.3)^*^	23 (63.9)^*^	3 (12.0)	3 (20.0)	6 (15.0)
PACI	26 (9.3)	15 (6.6)	41 (8.1)	106 (41.6)	104 (49.3)	210 (45.1)	20 (7.5)	16 (7.4)	36 (7.4)
LACI	1 (2.6)	0	1 (1.5)	10 (27.0)	10 (37.0)	20 (31.3)	1 (2.6)	3 (11.1)	4 (6.2)
POCI	24 (14.5)	9 (8.3)	33 (12.0)	43 (30.5)	35 (35.0)	78 (32.4)	17 (11.6)	6 (5.9)	23 (9.2)
Stroke severity:									
Mild	12 (3.8)^*^	8 (3.7)^*^	20 (3.7)^*^	74 (24.1)^*^	58 (27.5)^*^	132 (25.5)^*^	25 (8.0)	12 (5.6)^*^	37 (7.0)^*^
Moderate	30 (20.5)	8 (6.6)	38 (14.2)	71 (61.2)	77 (68.1)	148 (64.6)	11 (8.8)	10 (8.6)	21 (8.7)
Severe	22 (40.0)	14 (32.6)	36 (36.7)	29 (87.9)	23 (79.3)	52 (83.9)	5 (12.2)	6 (18.2)	11 (14.9)
Hypertension:									
Yes	45 (12.3)	24 (8.0)	69 (10.4)	126 (39.4)	124 (44.9)	250 (41.9)	30 (9.0)	25 (8.8)	55 (8.9)
No	19 (12.3)	6 (7.2)	25 (10.5)	48 (35.3)	34 (44.2)	82 (38.5)	11 (7.6)	3 (3.8)	14 (6.3)
Diabetes:									
Yes	13 (9.0)	10 (8.0)	23 (8.6)	56 (42.7)	52 (45.2)	108 (43.9)	9 (6.7)	8 (6.8)	17 (6.7)
No	51 (13.6)	20 (7.8)	71 (11.2)	`118 (36.3)	106 (44.5)	224 (39.8)	32 (9.3)	20 (8.2)	52 (8.8)
AF:									
Yes	8 (18.6)	1 (2.9)	9 (11.7)	11 (31.4)	17 (51.5)	28 (41.2)	3 (8.1)	5 (15.2)	8 (11.4)
No	56 (11.7)	29 (8.3)	85 (10.3)	163 (38.7)	141 (44.1)	304 (41.0)	38 (8.6)	23 (7.0)	61 (7.9)
Obesity:									
Yes	2 (3.6)^*^	4 (5.7)	6 (4.8)^*^	26 (49.1)	33 (50.0)	59 (49.6)^*^	5 (9.3)	6 (8.8)	11 (9.0)
No	62 (13.3)	26 (8.3)	88 (11.3)	148 (36.7)	125 (43.6)	273 (39.6)	36 (8.5)	22 (7.5)	58 (8.1)
Current smoking:									
Yes	17 (11.6)	4 (8.0)	21 (10.7)	55 (42.6)	14 (30.4)^*^	69 (39.4)	13 (9.5)	2 (4.3)	15 (8.2)
No	47 (12.6)	26 (7.8)	73 (10.3)	119 (36.4)	144 (46.9)	263 (41.5)	28 (8.2)	26 (8.3)	54 (8.2)
Alcohol drinking:									
Yes	6 (9.4)	0	6 (9.1)	21 (36.2)	0	21 (35.0)	6 (10.0)	0	6 (9.7)
No	58 (12.7)	30 (7.9)	88 (10.5)	153 (38.4)	158 (45.0)	311 (41.5)	35 (8.4)	28 (7.8)	63 (8.1)
TC									
Yes	4.72 (1.26)	5.24 (1.40)	4.89 (1.32)	4.61 (0.96)	5.25 (1.20)	4.91 (1.13)	4.41 (0.90)	5.23 (1.07)	4.62 (1.08)
No	4.54 (0.93)	5.20 (1.08)	4.82 (1.05)	4.50 (0.91)	5.16 (0.97)	4.77 (0.99)	4.55 (0.94)	4.95 (1.28)	4.84 (1.05)
TG									
Yes	1.18 (0.74)	1.30 (0.67)	1.22 (0.71)	1.17 (0.60)	1.53 (1.59)	1.34 (1.18)	1.15 (0.43)	1.35 (0.97)	1.23 (0.69)
No	1.23 (0.63)	1.45 (1.17)	1.32 (0.91)	1.26 (0.65)	1.38 (0.65)	1.31 (0.65)	1.23 (0.64)	1.44 (1.17)	1.32 (0.92)
HDL-C									
Yes	1.20 (0.67)	1.27 (0.40)^*^	1.23 (0.59)	1.14 (1.18)	1.14 (0.30)	1.14 (0.88)	1.02 (0.30)	1.13 (0.33)	1.06 (0.31)
No	1.08 (0.77)	1.14 (0.30)	1.11 (0.61)	1.05 (0.28)	1.15 (0.29)	1.09 (0.29)	1.09 (0.78)	1.15 (0.29)	1.12 (0.62)
LDL-C									
Yes	2.90 (0.82)	3.22 (1.35)	3.01 (1.03)	2.87 (0.77)	3.33 (0.98)	3.08 (0.90)^*^	2.69 (0.64)	3.05 (1.16)	2.83 (0.88)
No	2.82 (0.76)	3.24 (0.91)	3.00 (0.85)	2.79 (0.75)	3.17 (0.85)	2.95 (0.82)	2.83 (0.77)	2.26 (0.91)	3.02 (0.86)
FPG									
Yes	7.07 (2.92)	8.61 (3.94)^*^	7.54 (3.31)^*^	6.51 (2.03)	6.78 (2.85)^*^	6.64 (2.44)^*^	6.69 (2.07)	6.94 (2.60)	6.77 (2.23)
No	6.31 (2.31)	6.39 (2.50)	6.35 (2.39)	6.19 (2.47)	6.07 (2.13)	6.14 (2.34)	6.30 (2.33)	6.37 (2.49)	6.33 (2.40)

All data presented as *n* (%); ^*^ indicated *P* < 0.05 when comparing the outcomes between groups in men, women, and total.

OCSP: Oxfordshire Community Stroke Program; TACI: total anterior circulation infarcts; PACI: partial anterior circulation infarcts; LACI: lacunar infarct; POCI: posterior circulation infarcts; AF: atrial fibrillation; TC: total cholesterol; TG: triglycerides; HDL-C: high-density lipoprotein cholesterol; LDL-C: low-density lipoprotein cholesterol; FPG: fasting plasma glucose.

**Table 4:** Univariate analysis for risk factors of outcomes at 12 months after stroke both in men and in women

Factors	Mortality			Dependency			Recurrence		
	Men	Women	Total	Men	Women	Total	Men	Women	Total
Age:									
Yes	80.24 (3.97)	78.57 (3.10)	80.21 (4.13)^*^	79.58 (3.80)	79.00 (3.02)	79.12 (3.53)	79.97 (3.93)	78.83 (3.09)	79.50 (3.64)
No	79.45 (3.84)	79.09 (3.95)	79.21 (3.77)	79.36 (3.87)	79.10 (3.81)	79.24 (3.90)	79.81 (3.81)	79.03 (3.85)	79.24 (3.83)
OCSP:									
TACI	13 (43.3)^*^	7 (38.9)^*^	20 (41.7)	8 (47.1)	6 (54.5)	14 (50.0)^*^	5 (23.8)	4 (30.8)	9 (26.5)
PACI	42 (18.3)	28 (15.6)	70 (17.1)	73 (38.8)	62 (40.3)	134 (39.5)	54 (24.8)	42 (24.4)	96 (24.6)
LACI	2 (5.4)	0	2 (3.3)	7 (20.0)	5 (20.8)	12 (20.3)	7 (18.9)	4 (16.7)	11 (18.0)
POCI	28 (20.9)	12 (12.0)	40 (17.1)	34 (31.8)	29 (33.3)	63 (32.5)	33 (28.0)	19 (20.2)	52 (24.5)
Stroke severity:									
Mild	26 (9.8)^*^	13 (7.0)^*^	39 (8.7)^*^	60 (25.2)^*^	47 (27.3)^*^	107 (26.1)^*^	60 (23.3)	34 (18.8)^*^	94 (21.4)^*^
Moderate	32 (26.9)	16 (16.0)	48 (21.9)	46 (52.9)	40 (47.1)	86 (50.0)	29 (28.4)	22 (23.2)	51 (25.9)
Severe	27 (55.1)	18 (47.4)	45 (51.7)	17 (73.9)	15 (75.0)	32 (74.4)	10 (28.6)	13 (46.4)	23 (36.5)
Hypertension:									
Yes	58 (19.0)	33 (13.0)	91 (16.3)	91 (36.8)	84 (38.4)	175 (37.6)	70 (25.4)	61 (25.5)^*^	131 (25.4)
No	27 (21.3)	14 (20.0)	41 (20.8)	32 (31.7)	18 (31.0)	50 (31.4)	29 (24.4)	8 (12.3)	37 (20.1)
Diabetes:									
Yes	18 (14.9)	19 (17.3)	37 (16.0)	41 (39.4)	33 (35.5)	74 (37.6)	26 (23.2)	22 (21.2)	48 (22.2)
No	67 (21.5)	28 (13.1)	95 (18.1)	82 (33.6)	69 (37.5)	151 (35.3)	73 (25.8)	47 (23.5)	120 (24.8)
AF:									
Yes	9 (27.3)	2 (7.4)	11 (18.3)	6 (25.0)	14 (56.0)	20 (40.8)	5 (17.9)	11 (42.3)^*^	16 (29.6)
No	76 (19.0)	45 (15.2)	121 (17.4)	117 (36.1)	88 (34.9)	205 (35.6)	94 (25.6)	58 (20.9)	152 (23.6)
Obesity:									
Yes	5 (10.4)	7 (11.5)	12 (11.0)	21 (48.8)^*^	20 (37.0)	41 (42.3)	16 (33.3)	14 (23.7)	30 (28.0)
No	80 (20.8)	40 (15.3)	120 (18.6)	102 (33.4)	82 (36.8)	184 (34.8)	83 (23.9)	55 (22.4)	138 (23.3)
Current smoking:									
Yes	23 (18.4)	5 (11.1)	28 (16.5)	31 (30.4)	11 (27.5)	42 (29.6)	26 (22.0)	8 (19.0)	34 (21.3)
No	62 (20.2)	42 (15.1)	104 (17.8)	92 (37.4)	91 (38.4)	183 (37.9)	73 (26.4)	61 (23.3)	134 (24.9)
Alcohol drinking:									
Yes	8 (16.0)	0	8 (15.7)	9 (21.4)^*^	0	9 (20.9)^*^	9 (19.1)	0	9 (18.8)
No	77 (20.2)	47 (14.6)	124 (17.6)	114 (37.3)	102 (37.0)	216 (37.1)	90 (25.9)	69 (22.8)	159 (24.4)
TC									
Yes	4.62 (1.16)	5.50 (1.14)	4.86 (1.06)	4.64 (1.11)	5.16 (1.15)	4.87 (1.15)	4.61 (0.96)	5.24 (1.10)	4.86 (1.06)
No	4.55 (0.97)	5.15 (1.07)	4.81 (1.06)	4.49 (0.88)	5.16 (1.02)	4.79 (1.00)	4.50 (0.95)	5.18 (1.07)	4.81 (1.06)
TG									
Yes	1.21 (0.80)	1.42 (0.72)	1.30 (0.69)	1.23 (0.65)	1.54 (1.85)	1.37 (1.32)	1.25 (0.62)	1.37 (0.77)	1.30 (0.29))
No	1.09 (0.87)	1.44 (1.25)	1.33 (1.01)	1.23 (0.61)	1.38 (0.70)	1.30 (0.68)	1.22 (0.67)	1.46 (1.30)	1.33 (1.01)
HDL-C									
Yes	1.13 (0.59)	1.21 (0.35)	1.06 (0.29)	1.01 (0.27)	1.14 (0.28)	1.07 (0.28)	1.00 (0.26)	1.14 (0.29)	1.06 (0.29)
No	1.09 (0.87)	1.14 (0.29)	1.13 (0.72)	1.14 (1.06)	1.14 (0.30)	1.14 (0.82)	1.12 (0.94)	1.15 (0.31)	1.13 (0.72)
LDL-C									
Yes	2.89 (0.82)	3.46 (1.13)	3.08 (0.88)	2.96 (0.89)	3.28 (0.92)	3.10 (0.91)	2.93 (0.81)	3.32 (0.94)	3.08 (0.88)
No	2.85 (0.79)	3.22 (0.90)	3.00 (0.87)	2.79 (0.73)	3.19 (0.89)	2.96 (0.82)	2.81 (0.78)	3.23 (0.92)	3.00 (0.87)
FPG									
Yes	6.86 (2.68)	7.45 (3.30)^*^	6.61 (2.39)	6.55 (2.25)	6.59 (2.36)	6.56 (2.29)	6.64 (2.37)	6.56 (2.45)	6.61 (2.39)
No	6.28 (2.68)	6.37 (2.41)	6.26 (2.41)	6.15 (2.55)	6.27 (2.44)	6.20 (2.50)	6.22 (2.44)	6.31 (2.38)	6.26 (2.41)

All data presented as *n* (%); ^*^ indicated *P* < 0.05 when comparing the outcomes between groups in men, women, and total.

OCSP: Oxfordshire Community Stroke Program; TACI: total anterior circulation infarcts; PACI: partial anterior circulation infarcts; LACI: lacunar infarct; POCI: posterior circulation infarcts; AF: atrial fibrillation; TC: total cholesterol; TG: triglycerides; HDL-C: high-density lipoprotein cholesterol; LDL-C: low-density lipoprotein cholesterol; FPG: fasting plasma glucose.

In men, OCSP classification, stroke severity, obesity, and alcohol drinking were associated with both mortality and dependency at 3 and 12 months after stroke. Age, OCSP classification, stroke severity, the occurrence of atrial fibrillation (AF), and high-density lipoprotein cholesterol (HDL-C) and FPG levels were associated with mortality, dependency, and recurrence at 3 and 12 months after stroke in women (Table 3, Table 4).

### Determinants of stroke outcomes at 3 and 12 months after stroke

Stroke severity was a common predictor of poor outcomes at both 3 and 12 months after stroke. Moreover, male sex and the FPG level were predictors of mortality at 3 months after stroke among elderly patients. The corresponding relative risks (RRs) and 95% confidence intervals (CIs) were 2.21 (1.19, 4.09; *P* = 0.012) for male sex and 1.17 (1.07, 1.28; *P* < 0.001) for FPG. Older age was an independent risk factor for mortality at 12 months, with a RR (95% CI) of 1.06 (1.01, 1.11; *P* = 0.030). Compared to that for POCI classification, the risk of dependency at 3 months after stroke was 77% higher among patients with TACI classification, *P* = 0.009 (Table 5).

**Table 5:** Adjusted OR (95% CI) for risk factors of outcomes at 3 and 12 months after stroke overall

Characteristics	Mortality		Dependency		Recurrence	
	3 months	12 months	3 months	12 months	3 months	12 months
Gender:		—	—	—	—	—
Men	2.21 (1.19, 4.09)^*^	—	—	—	—	—
Women	1.00	—	—	—	—	—
Age	1.06 (0.99, 1.14)	1.06 (1.01, 1.11)^*^	—	—	—	—
OCSP:	—	—			—	—
TACI	—	—	1.77 (1.15, 2.73)^*^	1.16 (0.78, 1.71)	—	—
PACI	—	—	2.24 (0.787, 6.42)	1.29 (0.54, 3.10)	—	—
LACI	—	—	1.46 (0.69, 3.06)	0.60 (0.29, 1.23)	—	—
POCI	—	—	1.00	1.00	—	—
Stroke severity:					—	—
Mild	1.00	1.00	1.00	1.00	—	—
Moderate	4.95 (2.49, 9.85)^*^	2.97 (1.88, 4.97)^*^	5.46 (3.62, 8.23)^*^	2.64 (1.81, 3.)^*^	1.26 (0.72, 2.21)	1.30 (0.70, 2.41)
Severe	17.25 (8.06, 36.91)^*^	10.93 (6.40, 18.69)^*^	18.53 (6.98, 49.20)^*^	7.23 (3.47, 15.05)^*^	2.31 (1.12, 4.75)^*^	3.74 (1.60, 8.74)^*^
Obesity	0.37 (0.13, 1.04)	—	1.23 (0.72, 2.08)	—	—	—
Drinking	—	—	—	—	—	—
LDL-C	—	—	1.10 (0.87, 1.38)	—	—	—
FPG	1.17 (1.07, 1.28)^*^	—	1.05 (0.97, 1.14)	—	—	—

^*^ indicated *P* < 0.05 when comparing the association between outcomes and factors using multivariate logistic regression analysis; OCSP: Oxfordshire Community Stroke Program;TACI: total anterior circulation infarct; PACI: partial anterior circulation infarct; LACI: lacunar infarct; POCI: posterior circulation infarct; LDL-C: low-density lipoprotein cholesterol; FPG: fasting plasma glucose.

In men, stroke severity was a risk factor for both mortality and dependency at 3 and 12 months after stroke. Moreover, mortality at 3 months was 61% lower in patients with TACI classification compared to that for those with POCI classification and 79% lower in patients who were obese compared to that in those who were normal weight (Table 6).

**Table 6:** Adjusted OR (95% CI) for risk factors of outcomes at 3 and 12 months after stroke in men

Characteristics	Mortality		Dependency		Recurrence	
	3 months	12 months	3 months	12 months	3 months	12 months
OCSP:					—	—
TAPI	0.39 (0.20, 0.75)^*^	0.64 (0.36, 1.13)	1.30 (0.81, 2.10)	—		
PACI	1.28 (0.48, 3.39)	1.03 (0.39, 2.74)	2.29 (0.78, 6.77)	—		
LACI	0.18 (0.02, 1.42)	0.24 (0.05, 1.11)	0.97 (0.41, 2.30)	—		
POCI	1.00	1.00	1.00	—		
Stroke severity:					—	—
Mild	1.00	1.00	1.00	1.00		
Moderate	7.68 (3.71, 15.90)^*^	3.49 (1.94, 6.28)^*^	4.70 (2.96, 7.46)^*^	3.37 (2.00, 5.66)^*^		
Severe	14.97 (6.29, 35.5)^*^	10.33 (4.86, 21.94)^*^	20.04 (6.76, 59.44)^*^	7.96 (2.97, 21.28)^*^		
Obesity:	0.21 (0.05, 0.93)^*^		—	2.02 (1.02, 4.00)^*^	—	—
Alcohol drinking:	—		—	0.52 (0.23, 1.17)	—	—

^*^ indicated *P* < 0.05 when comparing the association between outcomes and factors using multivariate logistic regression analysis; OCSP: Oxfordshire Community Stroke Program; TACI: total anterior circulation infarct; PACI: partial anterior circulation infarct; LACI: lacunar infarct; POCI: posterior circulation infarct.

Similar to men, stroke severity was an independent risk factor for mortality, dependency, and recurrence at 3 and 12 months after stroke for women. Moreover, mortality at 3 months after stroke increased by 16% for each mmol/L increase in FPG level. However, mortality at 12 months after stroke was associated with hypertension and AF, with RRs (95% CIs) of 2.56 (1.13, 5.82; *P* = 0.024) for hypertension and 2.87 (1.22, 6.76; *P* = 0.016) for AF (Table 7).

**Table 7:** Adjusted OR (95% CI) for risk factors of outcomes at 3 and 12 months after stroke in women

Characteristics	Mortality		Dependency		Recurrence	
	3 months	12 months	3 months	12 months	3 months	12 months
Age	—	—	1.03 (0.95, 1.11)	—	—	—
OCSP:				—	—	—
TAPI	0.68 (0.21, 2.14)	1.39 (0.53, 3.68)	2.68 (1.36, 5.25)^*^	—	—	—
PACI	0.71 (0.10, 5.18)	1.98 (0.42, 9.43)	1.31 (0.27, 6.33)	—	—	—
LACI	—	—	2.41 (0.78, 7.50)	—	—	—
POCI	1.00	1.00	1.00	—	—	—
Stroke severity:	—	—	—	—	—	—
Mild	1.00	1.00	1.00	—	—	—
Moderate	1.60 (0.42, 6.11)	1.70 (0.61, 4.76)	6.75 (3.52, 12.91)^*^	2.30 (1.33, 3.97)^*^	1.58 (0.66, 3.78)	1.30 (0.70, 2.41)
Severe	8.89 (2.39, 33.11)^*^	7.47 (2.49, 22.38)^*^	12.95 (3.85, 43.55)^*^	7.71 (2.64, 22.50)^*^	3.72 (1.29, 12.73)^*^	3.74 (1.60, 8.74)^*^
Hypertension	—	—	—	—	—	2.56 (1.13, 5.82)^*^
AF	—	—	—	2.10 (0.88, 5.02)	—	2.87 (1.22, 6.76)^*^
Smoking	—	—	0.81 (0.33, 1.95)	—	—	—
HDL-C	2.52 (0.51, 12.42)	—	—	—	—	—
FPG	1.16 (1.01, 1.34)^*^	1.05 (0.91, 1.21)	1.06 (0.94, 1.20)	—	—	—

^*^ indicated *P* < 0.05 when comparing the association between outcomes and factors using multivariate logistic regression analysis; OCSP: Oxfordshire Community Stroke Program; TACI: total anterior circulation infarct; PACI: partial anterior circulation infarct; LACI: lacunar infarct; POCI: posterior circulation infarct. AF: atrial fibrillation; HDL-C: high-density lipoprotein cholesterol; FPG: fasting plasma glucose.

## DISCUSSION

This was a hospital-based study that explored sex differences in stroke outcomes and associated risk factors at 3 and 12 months after atherosclerotic stroke in patients aged 75 years and over in China. We found that mortality at 3 months after stroke was higher in men than in women. Stroke severity was an important risk factor for stroke outcomes, including mortality, dependency, and recurrence, at 3 and 12 months after stroke. Moreover, male sex and FPG level were determinants of mortality at 3 months after stroke, and the TACI stroke subtype was an independent risk factor for dependency at 3 months after stroke overall. However, the TACI stroke subtype and obesity were protective factors against mortality at 3 months after stroke in men, while TACI was a risk factor for dependency at 3 months after stroke in women. In women, FPG level was a predictor of mortality at 3 months after stroke, and hypertension and AF were associated with recurrence at 12 months after stroke.

Previous studies have shown significant sex differences in stroke outcomes, with women reported to have greater functional impairment at 3 and 12 months after stroke [19–21], but sex differences in mortality have not been observed [19]. Women have been reported to have greater mortality, recurrence, and dependency rates at 3, 6, and 12 months after stroke compared with those for men [22]. In contrast to these studies, we found that men had a higher mortality rate than women did (12.3% vs 7.8%) at 3 months after stroke. We observed no sex differences in dependence and recurrence rates in elderly Chinese patients at 3 and 12 months after stroke.

Severity and age are the strongest reported predictors of outcomes in the acute phase of stroke [23–25]. Stroke severity increased the 10-year mortality adjusted for hypertension, diastolic blood pressure, anticoagulant use on admission, diuretics use on discharge, and β-blocker use on discharge [26]. In the present study, the severity of stroke was a risk factor for mortality, dependency, and recurrence at both 3 and 12 months after atherosclerotic stroke in both men and women.

Poor outcomes were observed in patients with TACI, whereas more favorable outcomes occurred following partial anterior circulation infarct (PACI). There was a mortality of > 35% in patients with TACI and POCI [27]. In this study, TACI was associated with increased dependency at 3 months after stroke overall and in women, but TACI was a protective factor against mortality at 3 months in men. The mechanism of this reversed finding between men and women is unclear.

The association between hypertension and recurrence has been inconsistent. Previous studies have reported a positive association between hypertension and stroke recurrence [28, 29], but no such association was observed in another study [30].

The correlation between AF and outcomes in stroke patients has also been uncertain, but most previous studies indicated that AF was associated with higher mortality rates [31–33]. The 1-year risk of mortality in the AF group was 1.24-fold higher than that in the non-AF group in a previous study (*P* < 0.001) [34]. In the present study, there was a higher rate of recurrence among elderly female patients with hypertension and AF at 12 months after stroke.

A recent study reported that older women were more likely to have diabetes mellitus, hypertension, dyslipidemia, and heart disease than were older men [35]. In line with that study, FPG level was an independent risk factor of mortality at 3 months after stroke in women.

One hospital-based stroke registry in China showed that obesity was a protective factor for men at 36 months after stroke, with the risk of mortality reduced by 70% in obese men [36]. In this study, obesity was a protective factor against mortality at 12 months after stroke in men. The mechanism that causes this phenomenon remains unclear and needs further study.

There are several limitations in this study. First, this study was conducted in a single hospital, and the limited sample size reduces its generalizability. Second, all patients were from the stroke unit in the department of Neurology, the First Hospital of Jiamisi University. Patients who experienced fatal stroke and died before being hospitalized were not included in this study, which may have influenced the evaluation of outcomes. In addition, we did not collect information on medications used.

This was a hospital-based study that explored sex differences in outcomes and associated risk factors at 3 and 12 months after atherosclerotic stroke in patients aged 75 years and over in China. We found that mortality at 3 months after stroke was higher in men than in women. Stroke severity was an important risk factor for stroke outcomes, including mortality, dependency, and recurrence at 3 and 12 months after stroke. Moreover, male sex and FPG level were determinants of mortality at 3 months after stroke, and the TACI stroke subtype was an independent risk factor for dependency at 3 months after stroke overall. However, the TACI stroke subtype and obesity protected against mortality at 3 months after stroke in men, while TACI was a risk factor for dependency at 3 months after stroke in women. In women, FPG level was a predictor of mortality at 3 months after stroke, and hypertension and AF were associated with recurrence at 12 months after stroke. These findings suggest that it is crucial to control the primary risk factors individually by sex, especially regarding the management of hypertension and AF, in order to improve the secondary prevention of stroke among the elderly and to reduce the burden of stroke in China.

## MATERIALS AND METHODS

### Study population

This study used data from a stroke registry in the Department of Neurology, Jiamusi University First Hospital, China; the inclusion criteria for stroke patients have been described in a previous study [37]. Briefly, we prospectively collected data on the clinical characteristics and outcomes for all ischemic stroke patients who were admitted to the department between January 2008 and December 2013. Stroke events were defined according to the World Health Organization’s criteria, and stroke was confirmed in all patients by neuroimaging [38]. Patients who experienced transient ischemic attack were excluded from this study. Patients with atherosclerotic stroke aged 75 years and older were included in this study.

The ethics committee of Jiamusi University First Hospital approved the study, and written informed consent was obtained from all patients or their next-of-kin.

### Information collection

Data collection and outcome evaluation were performed by senior neurologists using standardized variable definitions and scores. Stroke subtypes, which were classified on admission, included TACI, PACI, lacunar infarct (LACI), and POCI, according to the OCSP criteria [39]. Stroke severity was categorized into three groups according to National Institutes of Health Stroke Scale (NIHSS) scores: mild (NIHSS score: ≤ 7), moderate (NIHSS score: 8–16), and severe (NIHSS score: ≥ 17) [19]. Conventional stroke risk factors, including hypertension, diabetes mellitus, AF, and hyperlipidemia, were defined as self-reported previous medical history, and obesity was defined as a body mass index ≥ 30 kg/m^2^. The NIHSS score and Barthel index (BI) were evaluated on admission and at discharge; the modified Rankin scale (mRS) was assessed on admission, at discharge, and at 3 and 12 months after stroke.

### Evaluation of outcomes

Outcomes included mortality, dependency, and recurrence rates at 3 and 12 months after stroke. Mortality was defined as all-cause cumulative death at the corresponding follow-up time point. Dependency was defined as an mRS score > 2 [20]. Recurrence was defined as all new-onset vascular events, including stroke, myocardial infarction, and venous thrombosis. Follow-up was implemented according to a predetermined procedure; the same senior neurologist collected data at 1 year and 3 years after stroke. Follow-up occurred for all patients in a face-to-face interview, except for those patients who were re-examined in their local hospitals, who completed follow-up by telephone.

### Statistical analysis

Continuous variables, including age, NIHSS, BI, mRS, TC, TG, HDL-C, LDL-C, and FPG, were presented as means with standard deviations (or as medians with ranges where appropriate) and were compared between groups using Student’s *t-*test or the Mann-Whitney *U* test. Dichotomous variables, including stroke subtypes, severity, risk factors, and outcomes during follow-up after stroke, were presented as number of cases (rates); the risk factors were compared between groups with the chi-squared test. Kaplan-Meier was performed to assess the sex difference in the mortality and recurrence rates. The relationships between associated factors and outcomes were assessed using logistic regression analyses and presented as unadjusted RRs with 95% CIs. The determinants of outcomes were evaluated by logistic regression analysis after adjustment for covariates (including stroke severity, stroke subtype, and risk factors) and were presented using adjusted RRs with 95% CIs. All statistical analyses were performed using SPSS version 19.0 (SPSS Inc., Chicago, IL), and a two-tailed *p* < 0.05 indicated statistical significance.

## Abbreviations

AIS: acute ischemic stroke
LAA: large artery atherothrombotic
SAO: small artery occlusion
CE: cardioembolic
OCSP: Oxfordshire Community Stroke Program
TC: total cholesterol
TG: triglycerides
LDL-C: low-density lipoprotein cholesterol
FPG: fasting plasma glucose
AF: atrial fibrillation
HDL-C: high-density lipoprotein cholesterol
RRs: relative risks
CIs: confidence intervals
POCI: posterior circulation infarcts
TACI: total anterior circulation infarcts
AF: atrial fibrillation
PACI: partial anterior circulation infarcts
LACI: lacunar infarct
NIHSS: National Institutes of Health Stroke Scale
BI: Barthel Index
Mrs: modified Rankin scale
